# An exploration of the impact of working in pairs on the dental clinical learning environment: Students’ views

**DOI:** 10.1111/eje.12780

**Published:** 2022-02-07

**Authors:** Anna Dargue, Charlotte Richards, Ellayne Fowler

**Affiliations:** ^1^ University Hospitals Bristol and Weston NHS Foundation Trust Bristol Dental Hospital Bristol UK; ^2^ Teaching and Learning for Health Professionals University of Bristol Bristol UK

**Keywords:** clinical environment, collaborative learning, dyads, informal learning, undergraduate, workplace learning

## Abstract

**Introduction:**

The aims of this study were to explore the undergraduate dental clinical students' experiences and perspectives of paired working in the clinical learning environment.

**Materials and methods:**

An interpretivist methodological approach with a socio‐cultural lens was used. A stratified purposeful sampling strategy was chosen. Students digitally recorded three audio‐diaries using Gibbs' cycle to guide reflection on collaborating clinically with a peer. 1:1 semi‐structured interviews were held using a topic guide. Inductive thematic data analysis was undertaken.

**Results:**

Eight participants were recruited. Main themes related to individual characteristics (motivation, professionalism, knowledge and experience) and relational features (feeling safe, attaching value, positive working relationships) that contributed to effective collaborative partnerships. The social setting is important for learning in the dental clinical environment. Benchmarking is used by students to motivate and reassure. Students learnt from their peers, particularly when they felt safe and supported and had developed good relationships. A lesser quality learning experience was highlighted in the assistant role.

**Conclusion:**

Paired working for clinical training was viewed mostly positively. Working with a variety of peers was beneficial and enabled development of interpersonal skills and professionalism. More effective collaborative learning partnerships were described when students felt they belonged and had affective support. Disadvantages of paired working were noted as reduced hands‐on experience, particularly for senior students and when working in the assistant role. Ground rules and setting learning goals to change the mind‐set about the assistant role were recommended. Emotional and practical support of students is needed in the clinical setting.

## 1 INTRODUCTION

The clinical learning environment for undergraduate dental students is a daunting one due to the unique nature of the course where they undertake irreversible treatments on patients. By the end of their degree, they must be capable of independent unsupervised practice in line with the General Dental Council (GDC) requirements of a ‘safe beginner’.^1^ This preparation for their future professional role is acknowledged as being stressful and is a challenge unlike anything students have faced before.^2^ A systematic review found that most dental students experience moderate levels of stress during their training and that clinical factors contributed to 64% of all sources of stress.^3^ Studies have highlighted that at certain stages of learning, dental students prefer help from their peers and peer collaboration is one way to manage, cope with and reduce stress.^2^, ^4^

Workplace learning makes an important contribution to vocational practice.^5^, ^6^ It relies heavily on experiential learning, and so learning and healthcare delivery are concurrent in clinical workplaces.^7^ Billett has highlighted that supported participation is key to learning in the workplace and is framed by a socio‐cultural perspective, where the workplace environment and the interactions with workers affect learning.^5^ Thus, the social and participatory nature of learning in workplaces is particularly important and individuals typically learn in collaborative situations.^8^ Relationships are seen as crucial in the workplace and can influence learning.^6^ A number of educational theorists support learning as a socio‐cultural phenomenon. Lave and Wenger, who studied apprenticeships, noted the progression of students through increasing participation in ‘communities of practice’.^9^ Bandura's social learning theory describes observational learning and role modelling of senior colleagues.^10^ More research has been undertaken examining medical students and their learning within clinical environments than for dental students, and these findings are supported by socio‐cultural theories and demonstrate that students learn by participating in practice in authentic workplaces.^11^, ^12^, ^13^, ^14^

The pairing of dental students when working together in the clinical setting was first described in 1975 in the USA.^15^ Then, it was described as an ‘innovative approach’ introduced to solve logistical and financial problems due to a lack of chairside assistants. A UK dental school survey of their students’ opinions of operator‐assistant pairs a quarter of a century later found that the majority enjoyed and felt they benefitted from working clinically in this way.^16^ Pairing dental students is said to be a tried and tested method to confer clinical skills in undergraduate dentistry, and the collaborative learning that results due to a shared, active experience, enhances learning.^16^

Undergraduate dental students are unique in healthcare education in that they share responsibility for providing dental treatment for a patient. This can be defined as collaborative learning where ‘two or more health professionals work together in order to learn cognitive, technical and non‐technical skills related to patient care’.^17^ There is limited literature available to assess the quantitative effects of collaborative clinical learning, and the quality is variable.^18^, ^19^ The effectiveness therefore cannot be assessed. The qualitative studies appear to show that collaborative learning has social benefits in terms of support, greater confidence and feedback.^16^, ^20^, ^21^ It is also noted that students report increasing frustration as they become more senior as pairing reduces their hands‐on experience.^16^, ^21^ Few studies have explored in depth the students’ view of paired collaborative learning in the clinical setting. Only one study is relevant to dentistry, but due to its design there is limited depth in the findings.^16^ Dental students are important stakeholders in their education and have been recognised as being overlooked.^22^

Several worldwide studies have asked dental clinical students their views of effective clinical learning experiences.^23^, ^24^, ^25^ All found that students value peer interactions where they learn together by discussing and sharing knowledge and experiences. They identify active collaborative learning as being beneficial.^23^, ^24^ Similar opinions have also been noted amongst pre‐clinical dental students.^26^ An international, expert working group highlighted that students’ view the encouragement of collaborative learning, team activities, cooperation and discussion amongst peers as contributing to a positive academic environment.^2^

Whilst research exists to examine the learning of medical students in the clinical environment, little exists related to dental students. Clinical dental education has been described as a complex exemplar of situated learning.^27^ In order to maximise and support student learning in the clinical setting, an understanding of how students learn at the chairside, as well as the impact of pairing students on learning, is vital. The aim of this study was therefore to explore the dental clinical students’ experiences and perspectives of paired working on the clinical learning environment and, if necessary, what could be done to support and improve this.

## 2 METHOD

The study was conducted at a teaching hospital in the south‐west of England with approximately 350 undergraduate students. Students start clinically treating patients at the end of year two. Students can be paired with a peer from their own year, or a student who is more senior/junior than them. However, there is variation across the dental specialties, in particular for learning restorative skills, where students are more commonly paired with a partner from their year who they stay with throughout all three clinical years. This supported the socio‐cultural lens for workplace learning in the clinical setting. An interpretivist methodology was used because learning is socially constructed by students with the likelihood of different realities. This approach aimed to understand the individual student's views of the clinical setting and generate rich and comprehensive data to understand the students’ perspectives of paired working.

**FIGURE 1:**
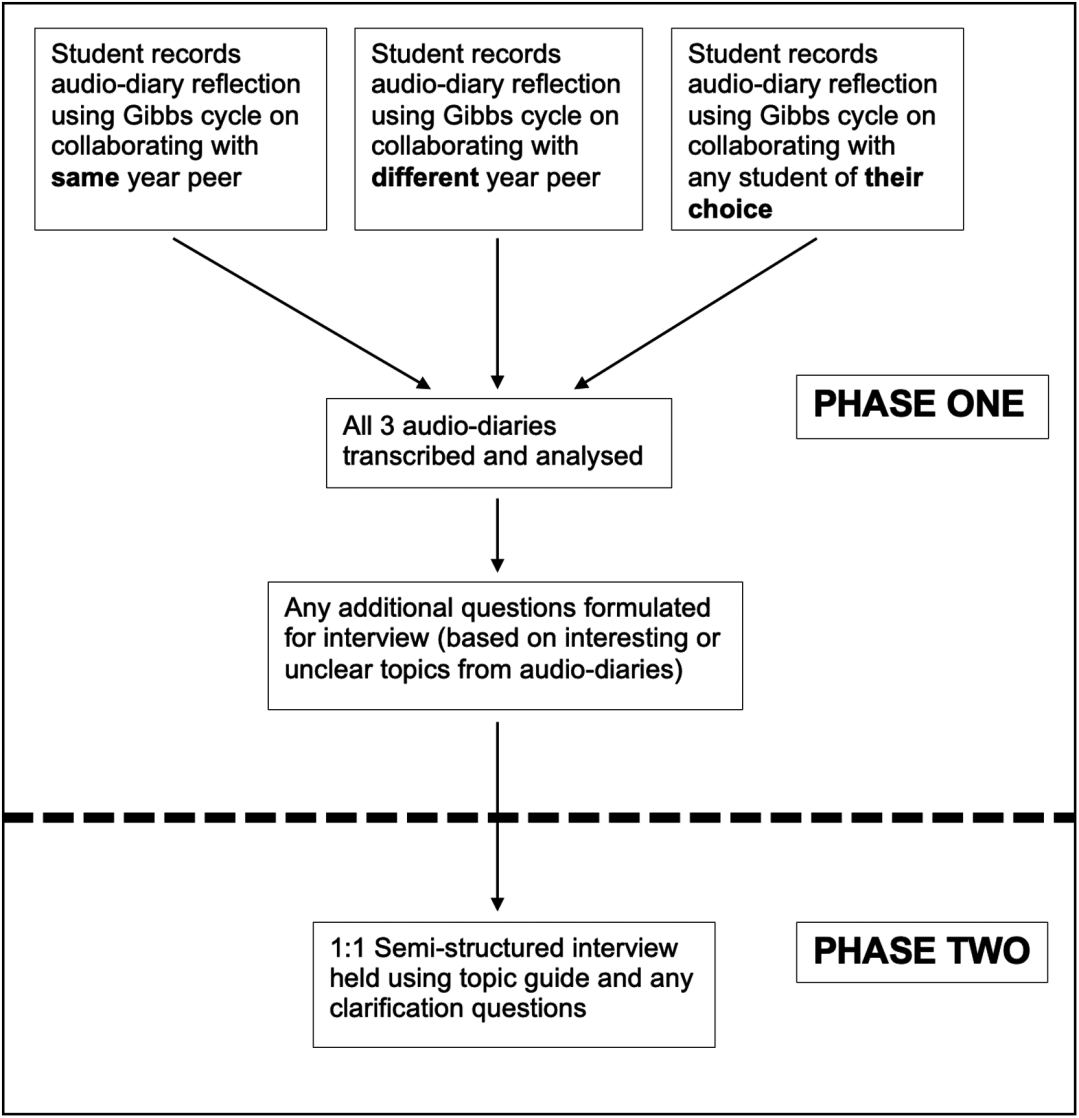
Flow chart to show data collection methods

A diary‐interview method was used to collect qualitative data. In phase one, students recorded reflective audio‐diaries followed by phase two, when a semi‐structured interview was conducted (Figure 1). A stratified purposive sampling strategy was chosen to recruit dental students in order to illustrate the characteristics of the different clinical years, highlighting the change in clinical experience across the three years and by comparing them, to identify how this might affect paired working.^28^ The aim was to sample ten to twelve students with a minimum of three students from each year. This is advised as necessary to enable comparison and to account for potential dropouts.^29^ Ethics approval was obtained from the university's research ethics committee. Participants were recruited from the third to fifth clinical years via email invitation using professional channels. Eight participants were recruited, and voluntary informed written consent was obtained from all students prior to the study commencing.

**FIGURE 2:**
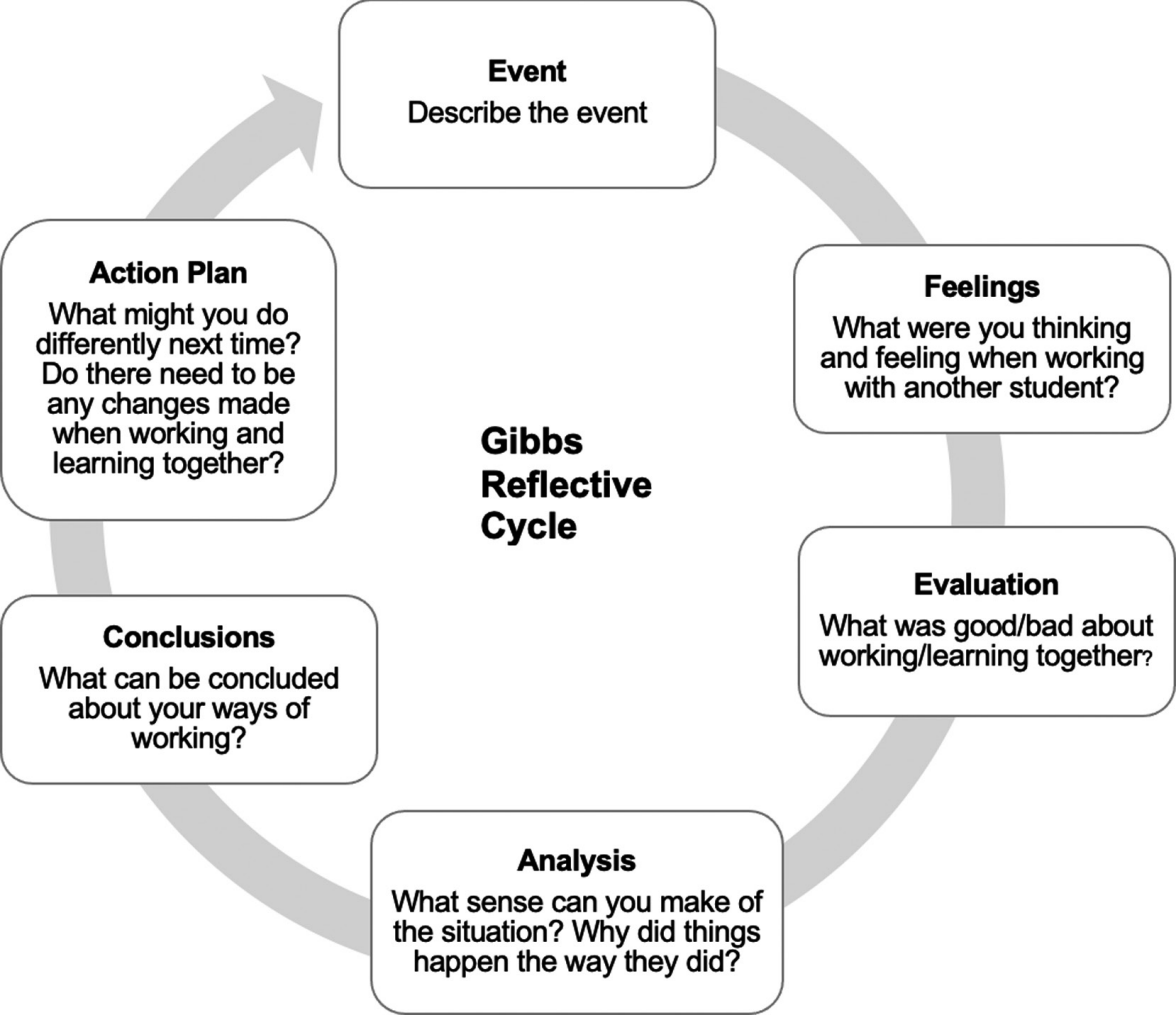
Question framework used by students to guide their reflection on paired clinical learning for the audio‐diary

In phase one, students were asked to record three 5‐min audio‐recordings with reflections on their experiences of a clinical session when working with a fellow student. They were asked to reflect on working with a student from the same year, a student from a different year and any student session of their choice (Figure 1). Students were encouraged to complete the recording as soon as possible after the clinical session. They were also asked whether they needed a suitable recording device; however, all had access to mobile phones that were able to digitally record their reflection. Email reminders were sent to students to encourage them to complete any outstanding recordings. Students were provided with a question framework, based on Gibbs’ reflective cycle, to guide their reflection on paired learning when completing their audio‐diary (Figure 2).^30^ Experts recommend using a framework to embed some structure and give consistency to responses and to reduce attrition rates.^31^, ^32^, ^33^ Gibb's cycle was chosen as it had been used successfully in similar studies with dental and dental hygiene students and has a straightforward structure with questions that encourage students to move through the six stages of reflection.^34^, ^35^ Students emailed their audio‐diary directly to the researcher, and the audio files were downloaded, anonymised, transcribed verbatim and then analysed. Any ideas that were unclear or areas of interest from the audio‐diaries were formulated into questions that were asked as part of the interviews in phase two. The time lag between the completion of the audio‐diaries and the interview was less than three months. The three audio‐diaries were emailed to students just prior to the interview to remind them of their reflections.

**FIGURE 3:**
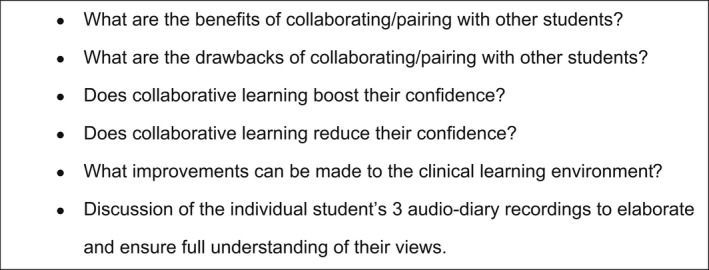
Topic Guide for Semi‐structured Interviews (Phase 2)

In phase two, a semi‐structured, one‐to‐one interview was carried out in a quiet, empty office of the dental school. A mutually convenient time and location were agreed. As the study took place over several months, from recording the audio‐diaries to attending the interview, participants were asked to verbally re‐confirm their consent prior to commencing the interview. They were reminded that the interviews were being recorded and that they had the ability to stop the interview at any time. The researcher is a part‐time clinical teacher at the dental school and so was known to the participants. She was also aware of the power dynamic in the relationship as both supervisor and researcher. To overcome this, a ‘romantic’ interview technique was used to establish genuine rapport by being friendly, open and honest with participants and by demonstrating a trusting and caring relationship.^36^ A topic guide was developed to guide the interviewer in the course of the interviews (Figure 3) along with follow‐up questions based on the participant's audio‐recordings. Every attempt was made to allow the participants to express their opinions without interruption or swaying their views. The digitally audio‐recorded interview was downloaded, anonymised and transcribed verbatim. The participants were emailed asking them to review their transcript and whether there were any alterations or areas they wished to expand on. Any suggestions or alterations were added to the transcripts.

Coding of the entire data set was completed after a process of familiarisation and immersion. Codes were collated into potential themes and reviewed both individually and across the data set. A data‐driven form of thematic analysis using an iterative process was performed to discover themes within the data.^37^ The researcher kept a diary, making reflections after each interview and during analysis, and this was used to promote reflexivity. The main themes were discussed with a ‘critical friend’, an experienced and trusted researcher who offered a sounding board and challenged ideas.

## 3 RESULTS

**FIGURE 4:**
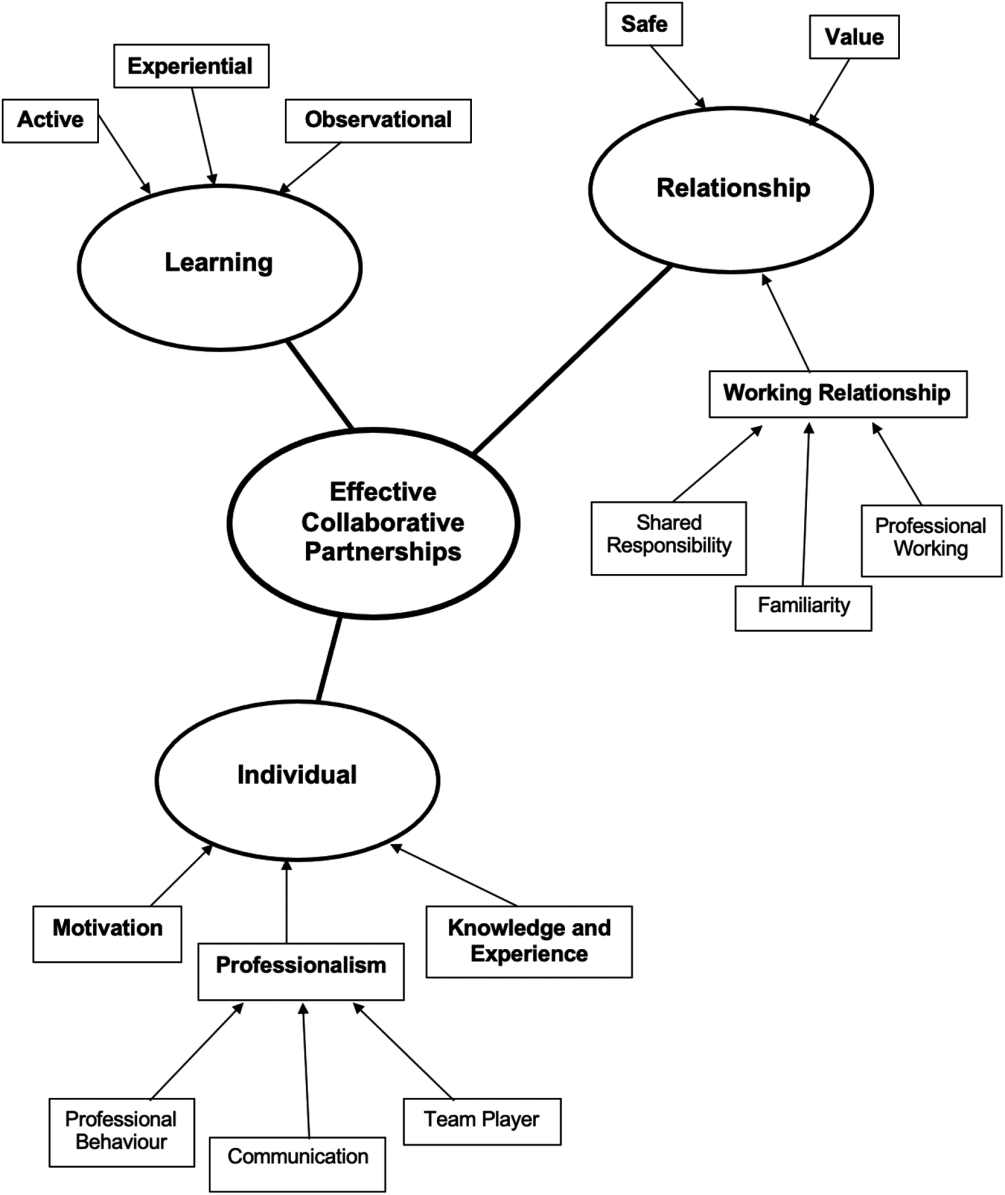
Diagram to summarise main themes for effective collaborative partnerships

Eight participants were recruited and completed the study: 3 final year students, 4 fourth‐year students and one third year student. There was one mature student and one male student. Students were viewed as junior students if they were the more junior student in the pairing and vice versa for senior students. This gave flexibility to fourth years in particular who could be either junior or senior, dependant on their clinical pairing. A summary of the student's role as operator or assistant in the pairing for each audio‐diary recording is shown in Table 1. The main themes that arose from the study exploring students’ experience of paired learning in the dental clinical setting related to factors that contributed to effective collaborative partnerships. Figure 4 summarises the main themes. Students identified active, experiential and observational learning occurring during effective student partnerships. These findings are not surprising and are reflected in the existing medical education literature related specifically to clinical dental students, as well as medical and nursing students.^4^, ^11^, ^13^, ^23^, ^25^, ^38^ This is not discussed further.

**TABLE 1:** Summary table to show the type of roles students were in when recording their audio‐diary reflection

Student year	Operator role	Assistant role	Both operator and assistant role
3rd year	1	2	0
4th year	3	8	1
5th year	7	1	1
Total number of audio‐diaries	11	11	2

The results that add to the dental educational literature featured participants describing individual characteristics that contributed to effective learning partnerships as well as the relational features of an effective working relationship. These themes are discussed further in this paper along with excerpts from the transcripts (audio‐diary (AD) and interview (I).

### 3.1 Individual student characteristics

Participants highlighted the importance of **motivation**. Students described positive feelings towards other students that were keen to learn and the beneficial effect this can have.

I do like people that are focused on their work…and gaining the most from the experience. (Year 4 AD)

A senior student emphasised that lack of engagement in their partner was a barrier to learning for both students.

Someone asked you a question… and it gets you thinking to answer the question, but that’s only if you get someone who cares enough to ask, and to be engaged in the session. (Year 5 I)

All student years described using ‘benchmarking’, a process for comparing their knowledge and abilities with that of their peers. For junior students working with senior students, this served to motivate them and reassured them that they too will become competent.

You get to see, and you get to also compare where you are to what they’re doing, and that’s someone to aspire to… you can compare yourself against them, yeah benchmark that’s the word. (Year 4 I)

Participants highlighted three aspects of **professionalism** that make students’ effective learners in their clinical partnerships: professional behaviour, good communicators and team players. Students exhibiting **professional behaviour** developed better relationships with their peers and described more effective learning. These included their partners being fair and honest which enabled them to share their learning experiences and to receive better quality feedback. They also highlighted other partner character traits such as being conscientious, polite and organised which contributed to a positive learning partnership.

We’re also fair to each other in order to make sure that we’re both gaining, um the same amount of experience without being unfair to the other person. (Year 4 AD)

We will always um thank each other um, at the end, especially if one of us has gone um, above and beyond, and we’ll also apologise if the situation does become stressful. (Year 4 AD)

Ethical behaviour such as empathy and caring for the welfare of fellow students and wanting to do their best for the patient were described as positive individual traits in both themselves and their colleagues. These professional behaviours contributed to better relationships between students and so support learning; they are also needed to provide quality patient care.

I know that when I’m working with anyone that I have to make sure that everything’s as good as it possibly can be for that particular patient, so whatever I think of that particular individual, I put it aside because at the end of the day I still need to give…good patient care. (Year 3 AD)

Students highlighted the importance of good **communication** between the partners as being vital for an effective learning partnership; it made them more efficient and work better as a team. Students identified learning communication skills from their peers during clinical sessions.

He made sure that he briefed me as to how he does things initially… being told what’s going to happen so I can anticipate what to do and what to get. (Year 3 AD)

I’ve definitely learnt ways in which I can communicate with the patient from fifth years. (Year 3 AD)

Students are likely to develop conflict at some point during their partnership so being able to communicate effectively to resolve this is vital.

What is good about working with my clinical partner is that we communicate really well, so that if we have any problems, we talk about it and that it’s resolved. (Year 4 AD)

Students in all years expressed that **working as a team** was part of being a professional and contributed to an effective learning partnership.

It’s good to learn how to work with other people, because that’s what you will be doing for the rest of your life. (Year 4 I)

Pre‐empting an operator’s needs is really important and can contribute to the speed and efficiency of anything getting done… I know how precious clinical sessions are… I think some assistants are really good at…thinking with you… (Year 5 I)

The relative levels of knowledge and experience of the individual students affected the partnership. Senior students described being more efficient and feeling comfortable teaching juniors, thus effectively collaborating.

It was quite nice to have someone who I felt like I could teach… because they haven’t really done endo on molars… and they don’t really know what perio‐endo lesions are yet, so um it was quite nice to explain everything. (Year 5 AD)

Junior students, however, described feeling frustrated with their lack of experience and knowledge and that this prevented them from supporting their colleague as effectively.

Um I don’t think anything went bad, um I just felt as if I was not as helpful as I could be, um because I didn’t have any previous experience with endodontic treatment…, so I felt that I wasn’t as efficient or as prepared for this session. (Year 4 AD)

The most junior student identified the learning she had made through collaborating in a partnership and recognised the important contribution she can make to teamwork and patient care.

I feel that from the year that I've had in assisting fifth years…originally I found it very challenging, very, very difficult. I didn't know where anything was. I found it quite embarrassing to constantly ask where things were, whereas now I feel I've seen the… the other side of it. So, I've not only improved in that I know where the materials are; I know why they're used, when they're used, so I can be really effective and efficient. (Year 3 AD)

### 3.2 Effective working relationships

Turning to the theme of relationships, students described features of a positive working relationship: feeling safe and valued, sharing responsibility for patient care, professional working and the importance of familiarity. All students across the three clinical years commented on the importance of feeling safe in their relationship which contributed to collaborative learning. The more established the relationship, the more comfortable they felt, and this encouraged more effective partnerships.

We were able to kind of discuss together and make the decision together about um what we were going to do, which I think sometimes if you’re not as comfortable or familiar with the person you are working with, you’re less inclined to do so as the assistant. (Year 5 AD)

Students described feeling comfortable asking questions of their peers, questions they felt less comfortable asking a tutor, and which helped them learn and develop.

I feel that sessions with fifth‐year students are very interactive and you can learn a lot from them and ask some questions that you might find quite difficult to ask, say a supervisor, because it could be, you know, deemed a silly question. So, it's actually a really good opportunity to just, just relax and ask questions and just figure out everything that you're not too sure about. (Year 3 AD)

Several students emphasised the emotional support that they both gave to, and received from, their partner. The following comment illuminates the supportive relationships that the students developed, and the encouragement and reassurance they gave each other.

My clinical partner definitely supports, because I get really stressed and she is always like calming me and you know supporting me and reassuring me. (Year 4 I)

Students also voiced feeling vulnerable when they felt they could not depend on their partners to support them.

This student just didn't care what we were doing, just was very uninterested…I just felt like I was literally…just I was thrown into something, I just had to deal with it, and it was just me on my own. (Year 5 I)

Students also discussed how they valued the efforts their partners went to in helping and supporting them.

I always try and say that if I have had a really good assistant, I say 'thank you so much, I couldn't have done it without you'…erm 'you know we really appreciate you being here'. (Year 5 I)

When I'm assisting and I know that I'm capable of…getting all the stuff for the operator and then they praise me…sometimes praise goes a long way. (Year 3 I)

The students valued both their colleague's advice and their feedback to support their learning.

We really value each other’s advice, and we don’t just ask for the sake of asking. We actually take it on board and just work it out. (Year 3 AD)

Students highlighted that more effective working partnerships have a shared aim; they shared responsibility for patient care. It also highlighted that the partners trusted and supported each other. This sharing of responsibilities involved discussing treatment options and contributed to their learning about the clinical case.

Decided it would be best to just call the patient in without the notes… while delegating um sort of responsibility to my clinical partner to find the notes…like I say I was happy to let my partner deal with the issue of finding the notes, and I would probably just say that as students we often rely on each other to help out. (Year 5 AD)

I think that as an assistant it shouldn't just be my job to like get stuff, I think as clinical partner's I think we should help each other out, um with… making clinical decisions and stuff. (Year 4 AD)

The transcript from this fourth‐year student's diary demonstrated the frequent use of subjective and possessive pronouns. This use of language highlights that she felt she shared responsibility for patient care, even though she was the assistant to a more senior student operator. This showed a professional desire to contribute to patient care.

So, **our** task was to…**we** tried to put… **we** decided to opt for…**we** wanted to give him time…the other options that **we** gave him. (Year 4 AD)

Professional working is similar to the subtheme of individual professionalism, but here it is applied to relationships. The students identified good communication and teamwork to be part of a professional working relationship and how working as a partnership is needed to achieve good patient care. Unprofessional partner behaviour was also described when students shared details of their partner's mistakes within the close‐knit dental student community. This lack of confidentiality can have a negative effect on confidence.

Actually, it kind of highlighted to me the importance of everyone working together for the same goal, otherwise at the end of the day the patient’s care suffers. (Year 5 AD)

I think the only very bad thing that can come out of working together is let's say someone makes a mistake, the entire Dental School will know within… that is probably a downside, that if people make mistakes, they're quite public. (Year 5 I)

They also recognised that having a good working relationship allowed them to feel comfortable giving and receiving useful feedback to support learning.

I find also with this particular clinical partner he um, is able to give me good feedback… and words of encouragement too. Um I’d like to think that I do the same for him, um and that he feels err the same in our working relationship. (Year 4 AD)

Students described the effects of increasing familiarity in their working relationship making their teamwork more efficient and so producing a more effective collaborative partnership. Several students highlighted that the opposite was also true.

We get so used to working with the same person, and you know exactly what they expect and how to work so it's very efficient. (Year 4 AD)

But when you work with somebody new it’s not that easy, I would say, because you have to kind of learn what they want, what they expect and so it’s not as um, I would say efficient. (Year 4 AD)

Significantly, students highlighted that only with increased familiarity and the development of a good working relationship, did their partners feel comfortable giving feedback for learning.

We are quite open so if one of us does something wrong during the appointment, we will just talk about it later…I think um initially she didn't feel comfortable, like being open with me, so telling me if I'd done something wrong, she felt awkward about it. (Year 4 I)

The students identified that friendships develop from the familiarity of their working partnerships and that this made them feel supported and produced a positive collaborative learning environment.

I was working with my er, 4^th^ year colleague, um who’s a very good friend… I’ve helped her with um choosing elective projects um….it was a very positive environment when we were working very well together it was quite humorous um and the patient was very relaxed considering the circumstances. (Year 5 AD)

### 3.3 Improvements to the clinical learning environment

Although the main thematic analysis explored students’ views and experiences of paired clinical learning, students were also asked their views on what could be done to improve the clinical learning environment. They suggested three areas for change that related to student partnerships: setting ground rules, clearer guidance about the assistant role and the benefit of changing clinical partners.

The students suggested ground rules for students to follow in the clinical environment would improve their partnerships and learning. Ground rules help students understand the expectations of them and their responsibilities. The students suggested covering several aspects of the previously discussed themes: professionalism and supportive working relationships. Students need to be advised of the need for professional behaviour when working as a clinical pair. This is highlighted from the following comment about sharing their peer's clinical ability with the wider dental student community.

I think there should be some sort of confidentiality law put in. (Year 5 I)

Students pointed out that feeling comfortable in their partnership allowed them to be more honest with giving, and more open in receiving feedback, as well as feeling comfortable to ask questions.

My and my clinical partner have this thing where when we see something interesting, we show each other and we just kind of learn from one another…we're good at sharing what we know with each other, so we learn faster. (5.2 AD)

The students suggested several changes could be made to clarify the assistant role in the partnership, as this was felt to offer a lesser learning experience. The changes surround creating a healthy learning environment where students are motivated to the benefits of being an assistant, encouraging participation in active and experiential learning, and demonstrating the value of this collaborative role. These changes link in with parts of the major themes already mentioned that of the individual and learning that contribute to effective collaborative partnerships.

The students suggested that they should be given guidance as to what the benefits are in assisting to help focus their experience and aid their reflection on learning.

I guess maybe at the start… they could sort of tell us why we're assisting and what they want us to get out of it so that when we go in, we kind of know what to do with ourselves…I guess it would be better if we could reflect on things that we had learnt more and kind of experienced… (Year 4 I)

The idea that the assisting student is encouraged to participate in the clinical session, so they are not only learning by observation, but also actively and experientially, was also suggested. The students proposed that both staff and students involve their assistant in the clinical session to make the learning experience a positive one and to maintain motivation.

I also think that changes can be made to make sure that the assistant is encouraged to participate almost in a dentist's role as well. So, for example…the assistant is encouraged to look at it, to feel it, hmm just so they, they can also gain more from the experience. (Year 4 AD)

The students suggested that there should be repercussions for assistants who do not attend their session. They felt this emphasised the value the dental school places on the assisting role and may contribute to a change in mindset by students on the importance of the assistant's contribution. Clinical dentistry provides most effective patient care if both an operator and an assistant are working together.

I mean I have one very practical thing, but I don't, it's basically that I feel that in assisting situations, a lot of students are getting away with not doing it…it would be nice if the people that did make the effort to assist got the benefit …I know they do because they get a lot of great learning experience blah blah blah, but I am sure at times they think, ‘why should I come and work in a pair because no‐one', like it doesn't really look like anyone else gets any repercussions from not… (Year 5 I)

Several students commented on the benefit of experiencing a variety of clinical partners. Currently, students have one clinical partner throughout the three clinical years in the department where they spend most of their time. The students suggested that it would be beneficial to change partners in this department, so they can learn teamwork and social skills and experience a variety of different viewpoints.

I think it's a good opportunity working with somebody else so that you learn to work with a whole array of people, um especially when we become dentists and work in the real world, we will have to work with a whole host of people and so it's a very good experience. (Year 4 AD)

## 4 DISCUSSION

The findings of this study support the importance of the social setting for learning in the dental clinical environment and so validate the methodological approach taken for this research. The advantage of combining both diaries and interviews, two well‐established methods of collecting qualitative data on a phenomenon, provides a richer data source with greater depth and clarity.^38^ Audio‐diaries are convenient to complete so reducing attrition rates and no dropouts occurred in this study.^32^ A significant advantage of audio‐diaries is hearing the students ponder and make sense of their experiences as well as being able to detect paralinguistic nuances, which add to the understanding of the words.^39^ Researchers use interviews to discover the individual's experiences and opinions.^40^ In our research, the diary‐interview method allowed the lead researcher to check the internal consistency of participants’ accounts by following up any gaps in the data and asking further questions.^33^

The students in this study gave a mostly positive view of paired working in the clinical setting. This reinforces findings from other studies that most dental students enjoy and benefit from working clinically with a peer.^15^, ^16^ Students in our study described the use of ‘benchmarking’. This technique was used to inspire, motivate and reassure that they would become competent and to identify any deficiencies in their knowledge/skills. A study by Lockspeiser et al refers to this benefit as ‘social congruence’, where students have similar social roles and provide valuable role models.^41^ It helps build confidence and offers reassurance as students can empathise with other students’ fears and anxieties, thus normalising the educational experience.^41^ Junior students in our study also highlighted the benefits of being partnered with a more senior student as they were more knowledgeable and experienced. They described learning from senior peers, and equally, senior students felt comfortable teaching their junior colleagues. The need for ‘legitimate’ role models in the eyes of the less experienced peer was reported by Roberts.^42^ This need for a competent or credible peer is reflected in the results of our study.

One of the key benefits of working with a student partner was that they felt safe, supported and comfortable working with their peers. They were able to discuss patient cases and ask questions they might otherwise feel unable to ask. A questionnaire on clinical pairings in dentistry corroborates this: students viewed the main advantages as being mutual help and support.^16^ Students in our study described the emotional support they received from their partners and valued this encouragement and reassurance. Emotional support in peer learning partnerships creates a comfortable learning environment.^43^ Dornan et al's review of how medical students learn in clerkships found that affective support was important to foster confidence and motivation to learn.^11^ Dornan et al's research is based on Billett's theory of ‘supported participation’, and our findings corroborate this research.^5^, ^11^, ^44^ Additional learning results from social interaction.

Another major benefit the participants identified from student pairings was the development of friendships where students had a good relationship with their clinical partners. A study examining peer learning experiences found that students valued friendships with peers as it made them feel part of a community and helped them cope with clinical practice.^42^ Students in this study described greater trust in established relationships, and this enabled them to give more honest feedback. Students are known to appreciate feedback that is provided in a positive emotional environment, and it is acknowledged as a significant contributor to successful learning.^45^, ^46^ Dental graduates must understand the role of appraisal and mentoring, and the importance of giving and receiving effective feedback as part of their professional role in the development of themselves and their dental team.^1^ Clinical partnerships are seen to offer students the chance to rehearse these skills.

As described above, the two main areas of benefit when working with a student partner were feeling safe and supported, and the development of good relationships and friendships. These areas correspond to two levels of Maslow's hierarchy of needs, the basic ‘safety needs’ and the psychological ‘belongingness and love needs’ and literature exists to support this.^47^ Several studies of nursing students during clinical placements suggest they need to feel they belong before learning can occur, as belonging influenced their capacity and motivation to learn.^48^, ^49^, ^50^, ^51^ For both nursing and medical students’, it has been noted that feeling they have a legitimate role in the clinical workplace has a social implication and an affective outcome on learning.^52^, ^53^ Similarly, a study of dental students identified that the ‘people’ environment is a powerful factor in engendering belongingness and developing collegiality with their peers (and staff) contributed to belonging.^54^ Thus, it appears that there is a great deal of evidence behind the students’ assertions that being part of a supportive relationship with their clinical partner, contributes in an affective way to a successful learning experience.

Students also identified that they developed their interpersonal skills through working with a variety of partners. Good social relationships are seen to maintain effective teams, so developing these skills as undergraduates is vital.^55^ Students noted that they learnt how to manage conflict with peers as part of expanding their communication skills. Students described trying to be efficient team members to contribute to quality patient care, thus modelling professional behaviours. A GDC guidance document on student professionalism reinforces that communication skills and effective teamwork are central to successful patient care.^56^

One of the objectives of the study was to establish what, if any, were the drawbacks of paired working in the clinical setting. Students in all clinical years revealed that observational learning was a lesser quality learning experience than experiential learning, that is there was a difference in learning between being the assistant versus the operator. Only more senior students highlighted their frustration and boredom in the assistant role, with two of three final year students commenting that being the assistant was less useful. The other final year student noted that junior students learn more than seniors. Two of the fourth years noted that being the assistant for a peer at their level was frustrating due to lack of learning. Only positive comments were made about the value of the assistant role by the most junior student. Thus, whilst the sample size is small, the research does strongly suggest that the assistant role offers less obvious learning.

Research examining effective learning experiences in undergraduate dentistry found that the use of modelling and demonstrations by observing a senior student was a valuable experience and Bandura's social learning theory supports this.^25^ However, students cannot progress through their community of practice without the opportunity to participate, which includes hands‐on practice, in order to become an experienced member of the community.^9^ A survey of medical students’ views on paired skills training found that students initially preferred being paired; however, as they become more experienced, they were concerned by reduced hands‐on practice.^21^ These findings mirror our own. Given that the undergraduate dental course's ambition is to produce independent students ready for clinical practice, a lack of experience in operative skills would be significant.^57^ Our research findings should serve as a stimulus for all dental schools, whilst pairing dental students during their early clinical experience is beneficial, for senior students it is less of an advantage. They need as much hands‐on practice as possible.

Students also described the tension they felt as the assistant having two roles: as both worker and learner. Junior students felt frustrated as their inexperience meant they were less effective at supporting their peer: senior students likewise noted that working with an inexperienced peer was less efficient, so less learning occurred. Parallels are seen with a survey of student's views on dental pairings, where senior students reported the disadvantage of working with a junior student due to reduced productivity and increased time pressure.^16^ Our study acknowledges the tension of learning in an authentic workplace. Students need to develop situation‐specific competences by participating, that is learning happens by doing the job itself.^8^ It is unsurprising that students identify this experience as challenging and stressful.^2^, ^3^ This should be considered when designing ways to support student learning in the dental clinical setting.

Interpersonal issues were noted as the other downside of paired clinical working. These related to lack of engagement and inappropriate communication. Students identified that being paired with a peer who lacked motivation created a barrier to learning as the student felt unsupported. Similarly, assisting students highlighted that they were not engaged if they did not understand the significance of their clinical role. So how can we motivate students and promote engagement? Setting strong, well‐determined learning goals to build engagement and enhance informal learning in the workplace is recommended.^5^ Clinicians need to stimulate learners to want to know and to value the outcome of the learning.^58^ Students are more engaged if their interests and values align with that of the workplace setting.^5^ Staff feedback on clinical work and progress can also help motivate.^59^ Dornan et al's research on medical students’ workplace learning illustrates a learning cycle where support builds motivation (and confidence) which enhances the student's sense of reward and identity and this then encourages further participation in order to learn.^44^ Support and belonging appear highly intertwined in the educational environment and highlight the importance of the social impact on learning.

Inappropriate communication, where students shared their partners’ mistakes with the wider student community, was highlighted as unprofessional behaviour and undermined the learning partnership. Authors researching clinical workplace learning highlight the need for trust in social interactions within the working environment.^6^, ^13^ Qualitative research on dental student's opinions emphasised that the opportunity to learn from each other's mistakes contributed to effective clinical learning experiences.^23^, ^24^ However, this can only happen in a space where trust and respect exist, creating a strong relationship that facilitates learning.^6^, ^60^ The students in our study corroborate these findings.

### 4.1 Improvements to the clinical learning environment

Participants in the study suggested three key recommendations to improve the clinical learning environment: changing partners, ground rules and learning culture. The advantages of working with a *variety of clinical partners* were recognised as experiencing different perspectives and exchanging ideas, facilitating the use of ‘benchmarking’ and learning interpersonal skills. Undergraduate students appreciated that interpersonal skills were important for their future working career.^61^ However, students moderated this recommendation noting that some stability in clinical partnerships was also valuable, due to the emotional support and effective working relationships that developed with familiarity. Thus, keeping clinical pairings for approximately one year, rather than staying in the same pairing for the three clinical years, was suggested by a consensus of students across all levels of experience.

Students also recommended that setting *ground rules* for pairings in the clinical environment would improve their partnerships and thus their learning. Students in the study proposed ‘professionalism’ and ‘effective working relationships’ as areas to discuss. Thus, undergraduate students already recognise the important contribution of professional behaviour and interpersonal skills to a collaborative clinical partnership. There is a paucity of evidence for the use of ground rules in the health professions literature and where discussed it relates to interprofessional education. Ground rules are recognised to help create a safe environment where students develop mutual trust and respect and can be used to contribute to a *learning culture*.^62^ Making a learning culture a high priority defines the attitudes, behaviour and practice of the setting.^63^

The assistant role in the student pairing was most discussed during the study as being a lesser quality learning experience. So, what can be done to improve student's learning experiences when in this clinical role? Setting learning goals for the assistant to engage and enhance learning would be beneficial. These could include cataloguing a range of behaviour management techniques used, to listing the treatment sequence for root canal treatment, and could even involve critiquing their partner's technical skills within a supportive learning environment. The possibilities are endless and can reflect the individual needs of the student. Similarly, encouraging staff and the operating student to allow the assistant to actively participate in the clinical session, encouraging as much learning as possible and supporting students to achieve their learning goals, should be promoted. As the students suggested, changing the mindset about the assistant's role is vital.

### 4.2 Limitations

A stratified purposive sampling strategy is suggested as appropriate when enough information is known about the phenomenon to identify characteristics that may influence how the phenomenon is manifest, and can lend credibility to the research.^64^ The lead researcher felt qualified to make this decision in her role as a clinical teacher, as well as with her involvement in the delivery and evaluation of the course. In reality, a convenience sample occurred as only eight students volunteered, so there was no choice in accepting the students; however, the lack of participants may have affected reliability. A pragmatic approach was taken to the research, although it is acknowledged that volunteer bias can be more significant in a convenience sample and can limit generalisability.^65^ It has been suggested that people who volunteer are more educated, are from a higher social class, are more likely to be female and are more motivated by approval.^66^ Although the proportion of female dental students is higher in UK dental schools (over 60%), there was still an over‐representation of female participants in our study.^67^ Again, this volunteer bias may have affected transferability.

To provide rigour in the research process, several accepted techniques were used to improve the trustworthiness, based on Guba and Lincoln's criteria for qualitative research (Table 2).^68^ The technique of ‘member checking’ or ‘respondent validation’ was carried out to allow students to corroborate, clarify or expand on their transcripts. This process is not without its criticism as participants may be defensive, censor, forget or wish to please the researcher.^64^ This is a possibility due to the main researcher's role as their clinical teacher. ‘Data triangulation’, where multiple perspectives of the same phenomena are analysed, was also used. A diary‐interview method allows data to be collected from different data sources at different times to develop a rich and comprehensive data set and allows other researchers the possibility to make judgements about the transferability of the study's findings.^31^ Data triangulation is directly linked to and ensures data saturation, and the research methods chosen were designed to achieve data saturation.^69^ Recall bias was minimised by asking participants to record each audio‐diary shortly after their clinical session as well as by emailing students their audio‐diaries to refresh their memories prior to the interview. Whilst recall bias cannot be eliminated, it was hoped that these techniques reduced any memory recall to its lowest level. The main researcher was a clinical teacher and had experience of learning in clinical partnerships, and this will have shaped her beliefs. However, whilst an insider's understanding can enrich and deepen research, it also brings a risk of subjectivity and bias, as well as a vested interest in the outcome.^70^ The personal lens of the researcher is also important in achieving data saturation, and here, the role of the ‘critical friend’ was used to discuss themes and mitigate bias in data collection.^69^ Being explicit about researcher bias promotes transparency in the research and allows an assessment of credibility and thus the importance of the research.^31^ It is hoped that this discussion highlights the efforts made in this study.

**TABLE 2:** Summary table to identify the techniques used to increase rigour in the research, based on Refs (31, 65, 68)

Trustworthiness	Parallel in quantitative research	Technique used in study
Credibility	Internal validity	Member checking Data triangulation Critical friend
Transferability	External validity	Thick description
Dependability	Reliability	Complete records Audit trail
Confirmability	Objectivity	Researcher reflexivity (diary)

Further qualitative research examining the difference in students’ views of paired working with same year and different year peers would be worthwhile as this study has only touched the surface of this topic. Further research into establishing and comparing students’ views of paired learning in different clinical settings alongside their clinical supervisors’ views would be beneficial. In addition, there is limited quantitative research on the effects of paired clinical learning, and nothing related to dentistry, so a large‐scale study at multiple dental settings would be of value.

## 5 CONCLUSIONS

Undergraduate dental students have a mostly positive view of being paired for clinical training. They described learning from their peers and their peers motivating them to learn. Working with a variety of clinical partners was seen as beneficial and helped develop their interpersonal skills and professionalism. Students highlighted the mutual social benefits of learning with a peer, in particular the affective support given to them in their clinical learning environment. They also emphasised that friendships developed in their partnerships which made them feel they belonged. Students, who felt safe and supported and belonged to their clinical partnerships, felt their collaborative learning partnership was more effective. This was because they were able to ask questions, felt able to request and receive honest feedback and were more confident.

The most significant disadvantage of pairing clinical students was noted in the frustration that students felt at having reduced hands‐on practice in the assistant role. Dental schools need to carefully consider how senior dental students are paired, perhaps using more dental nurses as the assistant, to increase hands‐on experience. Only sufficient hands‐on practice will allow the development of automaticity essential for independent practice. The other negatives of pairing also related to the assistant role in terms of the lack of engagement and the tension in this role as both worker and learner. The students recommended creating ground rules to improve the learning culture of the clinical setting. Ground rules create a safe learning environment where trust and respect in student partnerships are articulated through detailing the expectations, behaviour and responsibilities of their roles. Changing the mindset about the value of the assistant role could be achieved by setting learning goals. Similarly, promoting the need for active participation by the assistant to both staff and peers would help. Thus, the dental educational clinical setting needs to be organised to both, emotionally and practically, support student partnerships with their learning.

## CONFLICT OF INTEREST

None of the authors have any conflict of interest to declare.

## Data Availability

The data that support the findings of this study are available on request from the corresponding author. The data are not publicly available due to privacy or ethical restrictions.
